# BRIGHT Guidelines on Self-Monitoring of Blood Glucose 

**DOI:** 10.12669/pjms.305.6006

**Published:** 2014

**Authors:** Abdul Basit, Asima Khan, Riasat Ali Khan

**Affiliations:** 1 Abdul Basit, FRCP, Professor of Medicine, Department of Medicine, Baqai Institute of Diabetology and Endocrinology, Baqai Medical University, Karachi, Pakistan.; 2 Asima Khan, MBBS, Dip-Diab, Assistant Project Manager BRIGHT, Baqai Institute of Diabetology and Endocrinology, Baqai Medical University, Karachi, Pakistan.; 3 Riasat Ali Khan, MBBS, Dip-Diab, Project Manager BRIGHT, Baqai Institute of Diabetology and Endocrinology, Baqai Medical University, Karachi, Pakistan.

**Keywords:** Self Monitoring Blood Glucose, Diabetics, Guidelines

## Abstract

Pakistan, a developing country with limited resources, is having huge burden of diabetes and its complications. The local health care providers face limitations due to the related cost while emphasizing on self monitoring of blood glucose. The lack of health care infrastructure, non-affordability of the patients and non-existence of national guidelines are the most significant obstacles. Having realized these issues we decided to initiate a project of self monitoring of blood glucose, “BRIGHT (Better Recommendations, Implementation and Guideline development for Health care providers and their Training).

After extensive literature search, the project team, approached and communicated with “Advisory Board for the Care of Diabetes (ABCD) of Pakistan” for their expert opinion and suggestions. The board members belong to the faculty of main teaching hospitals of the four provinces of Pakistan thus ensuring national representation. The endorsement of these guidelines has paved the way for their uniform implementation all over the country.

Development of these Guidelines is the first part of BRIGHT project. In the next phase, we have started training of health care providers. Five mega programs have been conducted in this regard in the major cities. So far a patient’s log book has also been designed and distributed. Like all other guidelines, this is a living document which will be revised and updated from time to time in the light of new information which becomes available.

## INTRODUCTION

Diabetes mellitus is a well recognized health problem, the magnitude of which is increasing very rapidly. Currently, over 382 million people in the world have diabetes representing a prevalence of nearly 8.3%. Four out of five people with diabetes live in low and middle income countries. In half of people it remains unrecognized and half of the deaths in people from diabetes occur at an age under 60 years.^1^ The United Nations General Assembly unanimously passed a resolution (61/225) declaring diabetes “a global pandemic” affecting global health gravely and recognized it to be a debilitating, chronic and very expensive disease, especially if associated with complications.^2^ Many long term randomized controlled trials, in both type 1 and type 2 diabetes, have proved that intensive control of hyperglycemia significantly reduces the development of micro vascular complications.^3^^-^^7^ Long term follow up has proved that intensive glycemic control achieved in early stages of disease persists in terms of delayed development of macro-vascular complications even when degree of glycemic control in both intensive and control arm was comparable, an effect known as legacy effect.^8^ Better control of blood glucose requires appropriate monitoring. This makes self-monitoring of blood glucose an essential component of diabetes management. To ensure the effectiveness of self-monitoring, substantial diabetes education and an understanding of disease by people with diabetes themselves and by the health care providers is required. Self-monitoring provides an opportunity to document hyperglycemia or hypoglycemia, thereby allowing quick and optimal response to such situations without fear of over correction. The Diabetes Control and Complications Trial and other studies have proved that better metabolic control in adolescents and adults with type 1 diabetes is associated with fewer and delayed micro-vascular complications.^9^^-^^18^ It has also been established that self-monitoring of blood glucose on regular basis and its implementation in terms of dose adjustment of insulin along with carbohydrate intake and exercise is necessary to achieve optimal metabolic control.^19^^,^^20^ The topics of Self Blood Glucose Monitoring have been briefly covered in National Clinical Practice Guidelines for diabetes developed in 1999.^21^ There is now need to develop a comprehensive recommendation document for nearly 7 million diabetics in Pakistan considering our resource constraints.

Aims of self-monitoring of blood glucose include:

- To accurately assess level of metabolic control by individual therapy and achievement of realistic targets.^3^^,^^9^
- To assist in the prevention of both acute and chronic complications of diabetes.
- To reduce the effect of extreme glycemic conditions on cognitive function and mood of the individual.
- To assure proper data collection in various diabetes centers in order to provide an opportunity of comparison and improvement in interdisciplinary care for people with diabetes.^22^ These benefits can be attained by maintaining proper record either in a form of a diary or electronic record keeping. This record should include blood glucose readings, insulin dosage, record of special circumstances like illness, eating out, exercise, any episode of hypoglycemia and its severity and any episode of ketonuria or ketonemia.

**Better Recommendations, implementation and Guideline development for Health care providers and their Training (BRIGHT) [** Flow chart].

Following recommendations are proposed to guide people with diabetes and their healthcare providers in the use of Self Monitoring of Blood Glucose (SMBG).^23^

- SMBG recommendations would ensure that people with diabetes (and/or their care-givers) and their healthcare providers have the knowledge, skills and willingness to incorporate SMBG monitoring and therapy adjustment into their diabetes care plan, in order to attain agreed treatment goals.
- SMBG should be considered at the time of diagnosis to enhance the understanding of diabetes as part of individual’s education and to facilitate timely treatment initiation and titration optimization.
- SMBG should also be considered as part of ongoing diabetes self-management education to assist people with diabetes to better understand their disease and provide means to actively and effectively participate in its control and treatment, modifying behavioral and pharmacological interventions as needed, in consultation with their healthcare providers.
- SMBG protocols (intensity and frequency of SMBG) should be individualized to address each individual’s specific educational, behavioral or clinical requirements in order to identify, prevent and manage acute hyper- and hypoglycemia. The requirements of the care provider for collection of data on glycemic patterns and for monitoring the impact of therapeutic decision making should also be addressed.
- The purpose(s) of performing SMBG and using SMBG data should be agreed between the person with diabetes and the healthcare provider. These agreed-upon goals and actual review of SMBG data should be documented.
- SMBG requires an easy procedure for patients to regularly monitor the performance and accuracy of their glucose meter.
- Ketone test should be performed when needed, in type 1 individuals.
- In accordance with the sick day rule, the frequency of SMBG should be increased in special situations like fever, vomiting and persistent polyuria with uncontrolled blood glucose, especially if abdominal pain or rapid breathing is present (Table I-IV).

 **Setting Goals:**

In management of diabetes, optimal glycemic control is essential. Epidemiological studies clearly correlate uncontrolled diabetes with increased risk of micro and macro vascular complications regardless of management chosen.^24^^-^^26^ The risk of complications is related to both fasting plasma glucose and post prandial glucose levels with some evidence favoring postprandial glucose more strongly correlates with cardiovascular complications.^27^^-^^32^ In well resourced societies setting up targets for glycemic control is considerably easy. We have to consider our sub optimal resources both in terms of provision and expense of management. In these situations we can expect some degree of compromise either by health care provider or by individual himself, thus making target setting a difficult task. However pre set targets are fundamental to promote health care especially where prevention is main concern. There is hardly any randomized controlled trial designed to set glycemic targets. These targets are set by almost all leading organizations including ADA (American Diabetes Association)^33^^,^^34^, IDF (International Diabetes Federation)^35^, NICE (National Institute for Health and Care Excellence) type diabetes^36^, Canadian Guidelines^37^, and AACE (American Association of Clinical Endocrinologists)^38^ by expert consensus and based on epidemiological evidence for vascular complications associated with uncontrolled diabetes (Table-V). 

**Table-I:** BRIGHT recommended SMBG for lowest intensity

Twice weekly Days and timings are variable										
	*Breakfast*			*Lunch*			*Dinner*			*Bed Time*
	*Pre*	*Post*		*P* *re*	*Post*		*Pre*	*Post*		
										
Monday	♦									
Tuesday										
Wednesday										
Thursday										
Friday								♦		
Saturday										
Sunday										

*Controlled non affording type 2 diabetes*

*Women with controlled gestational diabetes on lifestyle modification*

*Geriatric patients (aged > 70 years) controlled with or without co-morbid conditions.*


* Is to decided according to the time of gestation*

**Table-II:** BRIGHT recommended SMBG for moderate intensity

* 1 – 2 Times Daily depending on control of blood glucose (7-14 points per week)* * Days and timings are variable*										
	*Breakfast*			*Lunch*			*Dinner*			*Bed Time*
	*Pre*	*Post*		*P* *re*	*Post*		*Pre*	*Post*		
										
Monday	♦						♦			
Tuesday		♦						♦		
Wednesday	♦			♦						
Thursday					♦					♦
Friday	♦						♦			
Saturday		♦						♦		
Sunday	♦									♦

*Newly diagnosed or uncontrolled non affording patients with type 2 diabetes *

*Controlled, affording patients with type 2 diabetes*

*Controlled, non affording patients with type1 diabetes*

*Women with uncontrolled gestational diabetes on lifestyle modification *

*Geriatric patients (aged > 70 years) uncontrolled with or without co-morbid conditions.*


* Is to decided according to the time of gestation*

**Table-III:** BRIGHT recommended SMBG for high intensity

*4 times daily (28 points per week)* * Days and timings are variable *										
	*Breakfast*			*Lunch*			*Dinner*			*Bed Time*
	*Pre*	*Post*		*P* *re*	*Post*		*Pre*	*Post*		
										
Monday	♦			♦			♦			♦
Tuesday	♦	♦			♦			♦		
Wednesday	♦	♦					♦			♦
Thursday	♦			♦	♦			♦		
Friday	♦	♦					♦	♦		
Saturday	♦			♦	♦					♦
Sunday	♦			♦			♦	♦		

*Newly diagnosed or uncontrolled affording patients with type 2 diabetes *

*Newly diagnosed or uncontrolled non affording patients with type 1 diabetes*

*Controlled affording patients with type 1 diabetes*

*Women with controlled gestational diabetes on insulin *

*Pre-operation care (duration of 1 week)*


*Is to decided according to the time of gestation*

**Table-IV:** BRIGHT recommended SMBG for high intensity

*4-6 times daily (28-42 points per week)* * Days and timings are variable *										
	*Breakfast*			*Lunch*			*Dinner*			*Bed Time*
	*Pre*	*Post*		*P* *re*	*Post*		*Pre*	*Post*		
										
Monday	♦			♦			♦			♦
Tuesday	♦	♦			♦		♦	♦		
Wednesday	♦	♦			♦		♦			♦
Thursday	♦	♦		♦	♦		♦	♦		
Friday	♦	♦		♦			♦			♦
Saturday	♦	♦		♦	♦		♦			♦
Sunday	♦			♦	♦		♦	♦		

*Newly diagnosed or uncontrolled affording patients with type 1 diabetes*

*Patients with type 1 and type 2 diabetes or gestational diabetes with inter-current illness or hospitalization *

*Women with uncontrolled gestational diabetes on insulin*

**Table-V:** Glycaemic targets

	***Sub category***	***Fasting blood sugar (mg/dl)***	***Random blood sugar (mg/dl)***	***Bed time blood sugar (mg/dl)***	***HbA1c (%)***
Gestational diabetes		65 – 90	70-120	110	< 6.0
Patients with type 2 diabetes	*Recent/without complications*	80 – 120	80 – 160	100 – 140	6.5 – 7.0
	*With CCF* * *, CKD* ” *, CLD* ≈ *, Autonomic neuropathy*	100 – 140	120 – 180	120 – 180	7.0 – 7.5
Patients with type 1 diabetes	*< 6 years*	100 – 150	100 – 250	100 – 200	8.0 – 8.5
	*6 – 12 years*	80 - 150	100 – 200	100 – 150	7.0 – 8.0
	*13 – 14 years*	80 – 140	100 – 180	100 – 120	6.5 – 7.0
	*> 14 years*	80 – 100	100 – 140	100 – 120	< 6.5

* *Congestive cardiac failure,*

” *Chronic kidney disease,*

≈ *Chronic liver disease*

**Figure:**
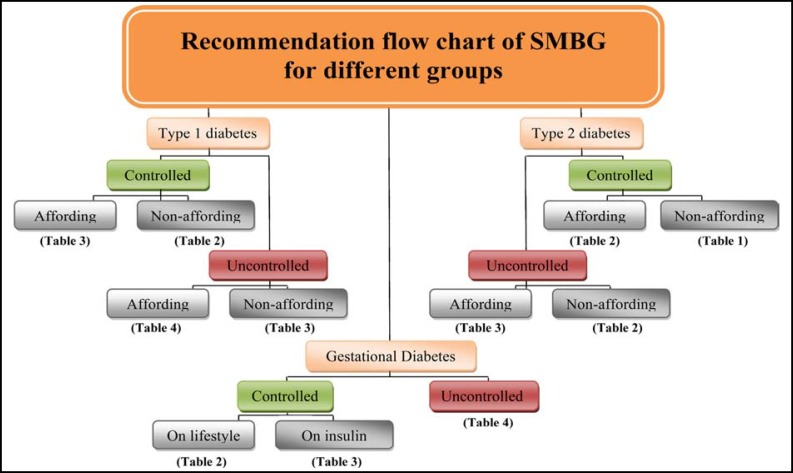
Flow chart

For special situations like Ramadan, Pilgrimage etc., we should set targets for our diabetic patients in accordance to their prior control, their expected excessive exercise, presence of complications, age of patient and weather conditions.
